# Evolving mortality rates in people who inject drugs: An Australian tertiary hospital observational study on infective endocarditis

**DOI:** 10.1371/journal.pone.0270283

**Published:** 2022-08-26

**Authors:** Isa Khan, Elizabeth Brookes, John Santamaria, Daniel Crisafi, Andrew Wilson, Jonathan Darby, Andrew Newcomb

**Affiliations:** 1 Melbourne Medical School, University of Melbourne, Melbourne, Australia; 2 Department of Cardiothoracic Surgery, St Vincent’s Hospital Melbourne, Fitzroy, Australia; 3 Department of Intensive Care, St Vincent’s Hospital Melbourne, Fitzroy, Australia; 4 Department of Cardiology, St Vincent’s Hospital Melbourne, Fitzroy, Australia; 5 Department of Infectious Diseases, St Vincent’s Hospital Melbourne, Fitzroy, Australia; 6 Department of Surgery, University of Melbourne, Melbourne, Australia; Case Western Reserve University School of Medicine, UNITED STATES

## Abstract

**Background:**

Injection drug use (IDU) associated infective endocarditis (IE) is clinically challenging due to social issues this population endures. Rates of IDU are rising globally, however, there is a lack of clear guidelines for IDU associated IE. The aim of this study is to assess the epidemiology of the IDU and non-IDU populations and compare their long-term outcomes to help guide future management.

**Methods:**

An observational cohort study was conducted on all 350 patients treated for IE at St Vincent’s Hospital Melbourne between 1999 and 2015. Follow up was performed until death or January 2021. Primary outcome was all-cause mortality.

**Results:**

IDU patients are younger (p<0.001), more likely to have concurrent infections (p<0.001), and other addiction disorders (p<0.001), while non-IDU patients are older with a higher level of comorbid illnesses (p<0.001). IDU and non-IDU patients received similar management during their admissions and experienced similar levels of in-hospital outcomes, except for non-IDU patient being more likely to develop pneumonia post-surgery (p = 0.03). IDU patients are more likely to become reinfected (p = 0.034) but have better long-term survival, with survival estimates at 15-years being 64.98% (95%CI: 50.94–75.92%) for IDU patients compared to 26.67% (95%CI: 19.76–34.05%) for non-IDU patients (p<0.001).

**Conclusion:**

Despite having higher levels of reinfection, IDU patients have better long-term survival compared to non-IDU patients. Therefore, we suggest IDU patients should not have blanket restrictions on the management they are offered unless at the individual level there is a contraindication to therapy.

## 1. Background

Despite infective endocarditis (IE) being a relatively rare disease with an annual incidence of 3 in 100,000 people globally, it is still associated with high mortality and morbidity [1–3]. Although in the contemporary era, older patients receiving medical intervention are most likely to be affected, it is younger patients who inject drugs that arguably provide the greatest clinical challenge [1–3]. Not only can the natural history of IE in the injection drug use (IDU) associated population be significantly different, making detection a diagnostic dilemma, social issues that burden this population can drastically change management, including suitability for surgery or long-term intravenous access for antibiotics [4–7]. Furthermore, IDU patients are likely to continue to use drugs after treatment, which has led some authors to argue that there should be restrictions on the level of intervention offered to IDU patients [8, 9]. Despite the prevalence of IDU associated IE set to increase due to rising rates of IDU globally, there is a lack of clear guidelines for this population in the current literature, which is leading to contention in regards to best practice. Our study aims to assess differences between the contemporary IDU and the non-IDU populations in regards to their presentation, management, and long-term outcomes [10]. Using these findings, we aim to help guide how to best manage IDU associated IE patients.

## 2. Methods

### 2.1. Study design and patient population

From April 1999 to January 2015, 350 patients accounting for 373 presentations were admitted to St Vincent’s Hospital Melbourne (SVHM) with IE. These patients were added to our institutional IE database. An observational cohort study was conducted and data for each patient was cross-referenced via hospital medical records, contacting current primary care physicians, the National Death Index, and the Australia New Zealand Society of Cardiac and Thoracic Surgeons (ANZSCTS) database. Data in the ANZSCTS database is collected using standardised datasets and definitions, and the data collection and audit methods have been previously described [11]. Patients were followed up until death or January 2021.

### 2.2. Definitions

IE was diagnosed according to the Modified Duke Criteria [12]. Medical management was defined as antibiotic therapy without surgery. Patients were initially commenced on empirical antibiotic regimens with rationalisation performed by Infectious Disease specialists once the aetiological organism was identified and antibiotic sensitivities determined. Patients were empirically commenced on combination intravenous Benzylpenicillin, Flucloxacillin, Ceftriaxone, and Vancomycin. Surgical management was defined as antibiotic therapy combined with surgical intervention. Relapse was defined as recurrence of IE within six months of the initial infection with the same organism. Reinfection was defined as recurrence of IE after six months of the initial infection or recurrence with a different organism. Intraoperative mortality was defined as death during surgery. Operative mortality was defined as death within 30 days of surgery. Other sites of infection are defined as infection of implantable cardiac devices or endocardium other than valve sites.

### 2.3. Variables and outcomes

The primary study outcome was all-cause mortality. The patient characteristics of interest assessed in this study were increasing age, sex, hepatitis B infection, hepatitis C infection, alcohol misuse, smoking, hypercholesterolaemia, hypertension, diabetes, coronary artery disease, New York Heart Association (NYHA) class, congenital heart disease, dialysis dependence, myocardial infarction, cardiac surgery, rheumatic heart disease, cerebrovascular disease, peripheral vascular disease, site of infection, aetiological organism, and surgery during admission.

### 2.4. Statistical analysis

Data was analysed with STATA IC version 15 (Stata Corp, College Station, TX, USA). Proportions data was summarised as total number and percentage, and continuous data was summarised as median and interquartile range (IQR). Continuous variables were compared using unpaired t-test. Categorical variables were compared using Chi-square test when the sample size was over five. Categorical variables were compared using Fisher’s exact test when the sample size was equal to or less than five. Long-term survival data was compared using Kaplan-Meier survival curves and log-rank test for equality.

### 2.5. Ethics statement

This study conforms to the ethical guidelines of the 1975 Declaration of Helsinki as reflected by the ethics approval for this study, designated QA 016/13, granted by the St Vincent’s Human Research Ethics Committee. De-identified data was collected and analysed anonymously; therefore the need for individual consent was waived.

## 3. Results

### 3.1. Cohort characteristics

A total of 350 patients accounting for 373 presentations were admitted for IE between 1999 and 2015 at SVHM. 79 of these patients were people who inject drugs. The IDU population was followed up for a median time of 9.30 years and the non-IDU population was followed up for a median time of 5.00 years. The baseline characteristics of these cohorts are summarised in Table 1.

**Table 1:** Patient characteristics. All categorical data is displayed as total number with percentage proportion in brackets. Continuous data is displayed as median with IQR in brackets. Statistical significance was determined using unpaired t-test for continuous variable, chi-square test for categorical variables with a sample size over five, and Fisher’s exact test for categorical variables with a sample size equal to or less than five.

	IDU *(n = 79)*	Non-IDU *(n = 271)*	p-value
**Demographics**			
Age (Years)	35.00 (27.00–42.00)	65 (51–74)	<0.001*
Male	55 (69.62)	186 (68.63)	0.87
**Comorbidities**			
Hepatitis B	7 (8.86)	3 (1.11)	<0.001*
Hepatitis C	56 (70.89)	2 (0.74)	<0.001*
Alcohol Use	25 (31.65)	32 (11.81)	<0.001*
Smoking	74 (93.67)	156 (57.56)	<0.001*
Hypercholesterolaemia	2 (2.53)	97 (35.79)	<0.001*
Hypertension	3 (3.80)	136 (50.18)	<0.001*
Diabetes	1 (1.27)	67 (24.72)	<0.001*
Coronary Artery Disease	2 (2.53)	77 (28.41)	<0.001*
Dialysis Dependence	0 (0)	26 (9.59)	0.004*
Congenital Heart Disease	13 (16.46)	47 (17.34)	0.85
Heart Failure (NYHA):			0.009*
Class 1	63 (79.75)	160 (59.04)	
Class 2	3 (3.80)	27 (9.96)	
Class 3	6 (7.59)	42 (15.50)	
Class 4	7 (8.86)	42 (15.50)	
**Past Medical History**			
Myocardial Infarction	1 (1.27)	30 (11.07)	0.02*
Previous Cardiac Surgery	2 (2.53)	99 (36.53)	<0.001*
Rheumatic Heart Disease	2 (2.53)	30 (11.07)	0.02*
Cerebrovascular Disease	10 (12.66)	57 (21.03)	0.10
Peripheral Vascular Disease	2 (2.53)	20 (7.38)	0.12
**Site of Infection**			
Aortic	26 (32.91)	129 (47.60)	0.06
Mitral	26 (32.91)	126 (46.49)	0.08
Tricuspid	36 (45.57)	21 (7.75)	<0.001*
Pulmonary	1 (1.27)	1 (0.37)	0.40
Multivalvular Endocarditis	15 (18.98)	37 (13.65)	0.24
Other site	9 (11.39)	33 (12.18)	0.85
Prosthetic Valve Endocarditis	2 (2.53)	56 (20.66)	<0.001*
**In Hospital Management**			
Length of Stay (Days)	27 (15–39)	26 (14–43)	0.31
Medical Management	50 (63.29)	174 (64.21)	0.88
Surgical Management	29 (36.71)	97 (35.79)	0.88

Assessing for level of comorbidity revealed that IDU patients were significantly more likely to have hepatitis B, hepatitis C, use alcohol, and smoke tobacco. The non-IDU population was significantly older, more likely to have hypertension, hypercholesterolaemia, diabetes, coronary artery disease, dialysis dependence, and present at a higher NYHA class. Furthermore, non-IDU patients were significantly more likely to have previously experienced a myocardial infarction, undergone cardiac surgery, and have had rheumatic heart disease.

Following echocardiography, it was found that IE was significantly more likely to affect the tricuspid valve in the IDU population and that prosthetic valve endocarditis was significantly more likely in the non-IDU population. Other sites were affected in similar proportions when comparing the two populations.

### 3.2. In-hospital management

The in-hospital management of this cohort has been summarised in Table 1. There were no significant differences in the proportion of patients receiving surgical intervention or medical management when comparing the IDU and non-IDU populations. There was also no difference between the overall lengths of stay between these two populations. Intraoperative details and postoperative outcomes have been summarised in Table 2. Analysis of surgical procedures performed found that there were no differences in the proportion of repairs versus replacements offered at each valve site between the IDU and non-IDU populations. Furthermore there were no significant differences in the proportions of complex patch procedures performed when compared the IDU and non-IDU populations. There were no significant differences in cardiopulmonary bypass time or cross-clamp time between the IDU and non-IDU populations.

**Table 2:** Operative details and postoperative outcomes. All categorical data is displayed as total number with percentage proportion in brackets. Continuous data is displayed as median with IQR in brackets. Statistical significance was determined using unpaired t-test for continuous variable, chi-square test for categorical variables with a sample size over five, and Fisher’s exact test for categorical variables with a sample size equal to or less than five.

Operative Details			
	IDU *(n = 29)*	Non-IDU *(n = 97)*	p-value
Aortic Replacement	21 (72.41)	62 (63.92)	0.40
Mitral Repair	5 (17.24)	21 (21.65)	0.59
Mitral Replacement	8 (27.59)	28 (28.87)	0.87
Tricuspid Repair	3 (10.34)	4 (4.12)	0.21
Tricuspid Replacement	2 (6.90)	0 (0.00)	0.05
Complex Patch Procedure	3 (10.34)	17 (17.53)	0.35
Cardiopulmonary Bypass Time (minutes)	147 (99–188)	158 (120–188)	0.24
Cross-Clamp Time (minutes)	114 (75–134)	119 (86–146)	0.30
**Postoperative Outcomes and Complications**			
Acute Myocardial Infarction	1 (3.45)	2 (2.06)	0.67
Cardiogenic Shock	5 (17.24)	27 (27.84)	0.25
New Atrial Fibrillation	4 (13.79)	26 (26.80)	0.15
New Ventricular Tachycardia	0 (0)	3 (3.09)	0.34
New AV Block Requiring Permanent Pacemaker	1 (3.45)	2 (2.06)	0.67
Stroke	1 (3.45)	3 (3.09)	0.92
Bleed	1 (3.45)	3 (3.09)	0.92
Prolonged Intubation (>24 hours)	8 (27.59)	33 (34.02)	0.52
Pneumonia	0 (0)	14 (14.43)	0.03*
Sternotomy Infection	0 (0)	1 (1.03)	0.583
Multiorgan Failure	4 (13.79)	9 (9.28)	0.48
Postoperative Haemofiltration	2 (6.90)	10 (10.31)	0.58
Intraoperative Mortality	4 (13.79)	9 (9.28)	0.71
Operative Mortality	5 (17.24)	12 (12.37)	0.60
Reoperation	4 (13.79)	9 (9.28)	0.48
Reinfection requiring reoperation	2 (6.90)	2 (2.06)	0.27
Time to Reoperation (Years)	4.34 (2.37–9.56)	8.32 (4.44–11.68)	0.49

On follow up, we found there were no significant differences in the need for reoperation following in the index surgery, nor the length of freedom from reoperation between the IDU and non-IDU populations. There were no significant differences in the proportion of postoperative complications between the IDU and non-IDU populations, except the non-IDU population was significantly more likely to experience pneumonia following surgery (p = 0.03).

### 3.3. Causative organisms

The causative organism in each presentation has been summarised in Table 3. The study found that methicillin susceptible Staphylococcus *aureus* (MSSA) was the most common aetiological pathogen in both the IDU and non-IDU groups. Furthermore it was found that non-IDU patients are associated with a higher proportion of Enterococcus *faecalis* infections.

**Table 3:** Aetiological organism. All characteristics have been displayed as total number with percentage proportion in brackets. Statistical significance was determined using chi-square test for categorical variables with a sample size over five and Fisher’s exact test for categorical variables with a sample size equal to or less than five.

	IDU *(n = 79)*	Non-IDU *(n = 271)*	p-value
Methicillin susceptible Staphylococcus *aureus*	47 (59.49)	95 (35.06)	<0.001*
Viridans Streptococci	13 (16.46)	38 (14.02)	0.59
Enterococcus *faecalis*	2 (2.53)	31 (11.44)	0.02*
Coagulase negative Staphylococcus	1 (1.27)	12 (4.43)	0.31
Methicillin Resistant Staphylococcus *aureus*	1 (1.27)	11 (4.06)	0.23
Enterococcus faecium	0 (0)	8 (2.95)	0.12
Streptococcus gallolyticus	0 (0)	6 (2.21)	0.18
Streptococcus *agalactiae*	1 (1.27)	5 (1.85)	0.73
Streptococcus anginosus	0 (0)	4 (1.48)	0.58
Abiotrophia *defectiva*	1 (1.27)	3 (1.11)	1.00
Actinobacillus actinomycetimcomitans	0 (0)	3 (1.11)	0.35
Group G Streptococcus	0 (0)	3 (1.11)	1.00
Streptococcus salivarius	0 (0)	3 (1.11)	1.00
Coxiella *burnetii*	1 (1.27)	2 (0.74)	1.00
Streptococcus pyogenes	1 (1.27)	2 (0.74)	0.54
Haemophilus aphrophilus	0 (0)	2 (0.74)	1.00
Streptococcus pneumoniae	1 (1.27)	2 (0.74)	0.54
Propionibacterium *acnes*	0 (0)	2 (0.74)	1.00
Candida species	2 (2.53)	1 (0.37)	0.07
Abiotrophia *adiacens*	1 (1.27)	1 (0.37)	0.40
Staphylococcus lugdunensis	0 (0)	1 (0.37)	1.00
Proteus mirabilis	0 (0)	1 (0.37)	1.00
Clostridium species	0 (0)	1 (0.37)	1.00
Aspergillus terres	0 (0)	1 (0.37)	1.00
Serratia marcescens	0 (0)	1 (0.37)	1.00
Scedosporium apiospermum.	0 (0)	1 (0.37)	1.00
Aerococcus urinae	0 (0)	1 (0.37)	1.00
Capnocytophaga species	0 (0)	1 (0.37)	1.00
Klebsiella pneumoniae	0 (0)	1 (0.37)	1.00
Haemophilus *parainfluenzae*	1 (1.27)	0 (0)	0.23
Streptococcus dysgalactiae	2 (2.53)	0 (0)	0.05
Organism Not Isolated	4 (5.06)	26 (9.59)	0.21

### 3.4. Outcomes and follow up

**Fig 1:**
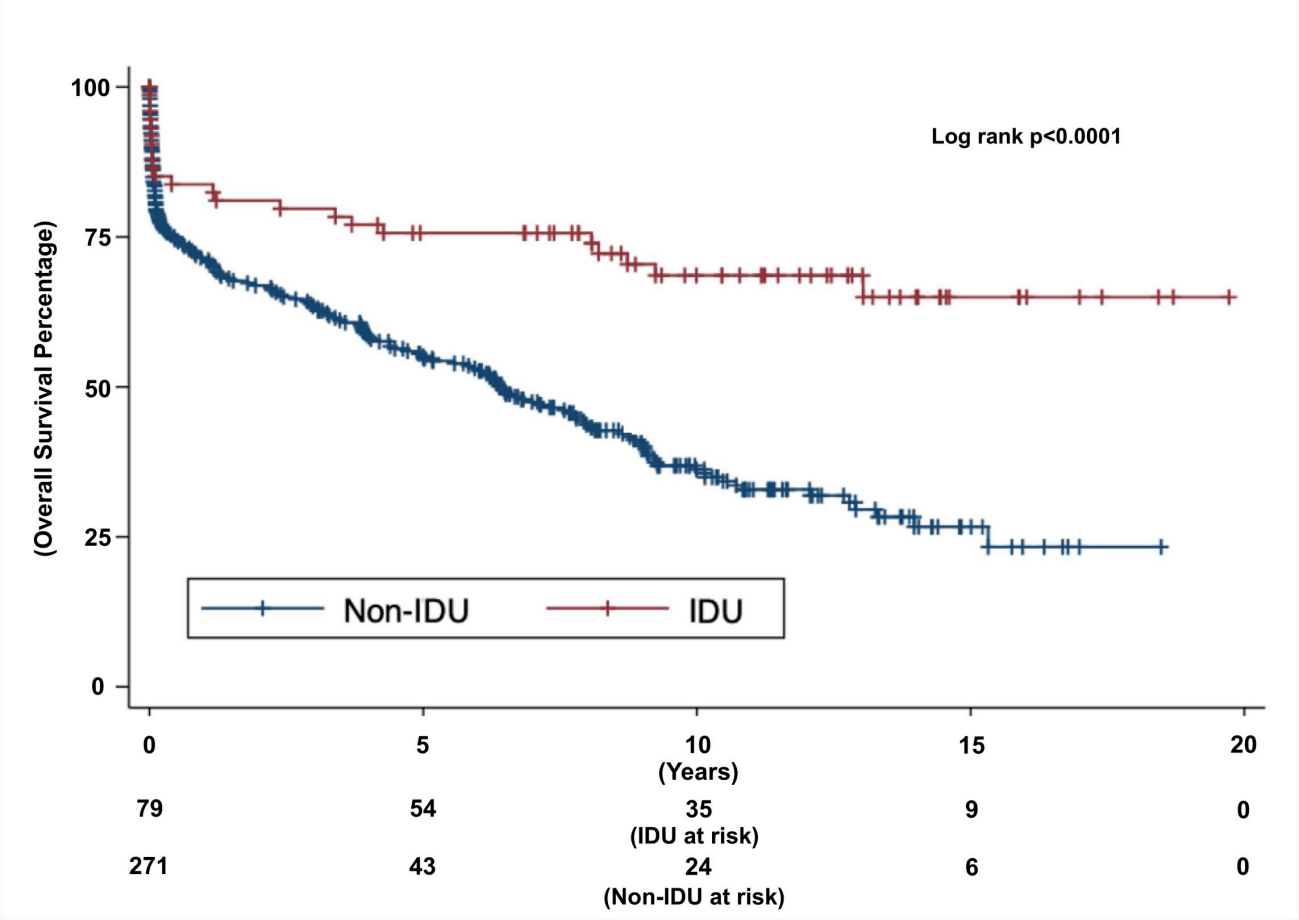
Injection drug use associated versus non-injection drug use associated Kaplan Meier survival curves. Light coloured line represents injection drug use associated populatuion and dark coloured line represents non-injection drug use associated populatuion. Y-axis represents overall survival in percentage. X-axis represents follow up length in years. Under the X-axis is the number of patients at risk in the IDU and non-IDU populations respectively. Censor bars indicate patient’s total follow up length without death.

The survival and follow up data of this study have been summarised in Table 4. IDU patients were significantly more likely to become reinfected compared to the non-IDU population. At the conclusion of this long-term study, overall mortality was found to be 29.11% in the IDU subpopulation compared to 61.99% in the non-IDU subpopulation (p<0.001). Kaplan-Meier survival analysis was used to assess long-term mortality, as depicted in Fig 1. Survival estimates were 83.78% at 1-year in the IDU population compared to 71.54% in the non-IDU population, 75.65% at 5-years in the IDU population compared to 55.16% in the non-IDU population, 68.59% at 10-years in the IDU population compared to 36.25% in the non-IDU population, and 64.98% at 15-years in the IDU population compared to 26.67% in the non-IDU population (p<0.001).

**Table 4:** Outcomes and follow up. All results except follow up length have been displayed as total number with percentage proportion in brackets. Follow up length has been represented as median with IQR in brackets. Mortality rates have been displayed as number of deaths with percentage proportion of at risk population for time period in brackets. Statistical significance was determined using unpaired t-test for continuous variable, chi-square test for categorical variables with a sample size over five, and Fisher’s exact test for categorical variables with a sample size equal to or less than five.

	IDU *(n = 79)*	Non-IDU *(n = 271)*	p-value
**Follow Up Rates**			
Lost to Follow Up	5 (6.33)	10 (3.69)	0.31
Overall Follow Up	74 (93.67)	261 (96.31)	0.31
Follow Up Length (Years)	9.30 (4.27–13.03)	5.00 (0.19–9.29)	0.55
**Relapse/Reinfection**			
Reinfection	9 (11.39)	13 (4.80)	0.03*
Relapse	1 (1.27)	0 (0.00)	0.06
**Mortality Rates**			
Overall	23 (29.11)	168 (61.99)	<0.001
1-year	1 (3.45)	5 (5.15)	
5-year	5 (17.86)	13 (13.83)	
10-year	12 (19.05)	8 (14.29)	
15-year	6 (11.11)	8 (18.60)	

## 4. Discussion

Injection drug use is a known risk factor for mortality [13]. Propensity matched studies are useful for assessing individual risk factors by limiting confounding, however, when assessing the overall outcomes of a population, all significant factors, including protective factors, should be included in the analysis. Our epidemiological study found that non-IDU patients are significantly older compared to the IDU population and are more likely to have significant comorbid illnesses. IDU patients are more likely to have coinfections and be affected by other addiction disorders. Previous studies regarding level of comorbidity in the IDU versus non-IDU populations are limited to surgical patients, thus our study is the first to show that a significant difference in the level of comorbidity extends to the overall IDU versus non-IDU populations [14]. Furthermore, a previous study conducted on IE patients between 1980–2004 found that IDU patients were more likely to present later in the IE disease course based on stage of heart failure [5]. Our study is the first to show that in the current era, IDU patients are likely to present at earlier stages of heart failure compared to the non-IDU population.

In our study, we found that IDU patients were more likely to have tricuspid valve involvement compared to the non-IDU population. Tricuspid valve IE is difficult to diagnose based on clinical signs and often not identified until there are complications [6]. This study adds our centre’s contemporary experience to the current body of evidence for clinicians to have a low threshold to suspect tricuspid IE in IDU patients.

In both cohorts, it was found that Staphylococcus aureus was the most common aetiological organism, which is consistent with the current literature [1]. It was also found that non-IDU patients are more likely to have an Enterococcus faecalis infection compared to the IDU population. This finding shows in the current era, older non-IDU population are at a significant risk of developing enterococci infections [15].

The primary aim of this study was to compare the long-term mortality of the IDU and non-IDU associated IE populations in the current era. We found that IDU patients today have better long-term survival compared to the non-IDU population. This finding is strengthened by the fact that there were no significant differences in the proportion or types of surgical intervention offered to each group and that the length of admissions for each population were similar. IDU patients were also significantly less likely to develop pneumonia following surgery (p = 0.03). This finding reflects an emerging change in the survival of IDU patients. Pericàs et al. recently published a large multinational observational cohort study that found IDU patients had lower in-hospital and 6 month mortality compared to non-IDU patients [16]. Our study builds on this finding and is the first to show that improved outcomes extend to long-term survival as well. The improved survival in the IDU population is most likely a reflection of the younger age and lower comorbidity level at time of presentation with IE, which are statistically significant protective factors associated with this population.

On follow up, it was found that 11.39% of IDU patients became reinfected and are statistically more likely to become reinfected compared to non-IDU patients. This is the first study to show that IDU patients are significantly more likely to become reinfected when directly compared to non-IDU patients [8]. Despite the higher level of reinfection in the IDU population, at our centre, there was not a statistically significant difference in whether reoperation was offered between the IDU and non-IDU groups.

The overall aim of this study was to help guide the management of IDU associated IE patients. A lack of clear guidelines in the current literature has led to contention regarding whether there should be differences in the treatment options available to IDU versus non-IDU patients. Hayden and Moore’s qualitative study on attitudes towards repeat surgery for patients who continued to inject drugs found that implicit and explicit biases remain in the current era against this population and that lack of clear guidelines can lead to inequitable care [17]. Despite ethical analyses outlining that IDU patients, as an overall population, should not have restrictions on their ceiling-of-care, some care providers argue that IDU-associated IE patients should not be offered endless rounds of expensive, high-risk interventions if they have reinfections caused by continued drug use on the basis that medical resources need to be fairly distributed and that futile treatments burden the rest of society [9, 18]. On follow up, it was found that 11.39% of IDU patients in our study became reinfected and are statistically more likely to become reinfected compared to non-IDU patients. Despite the higher level of reinfection in the IDU population, we found there was no statistical difference between IDU and non-IDU patients in regards to being offered reoperation. While there is no doubt that addiction therapy and cessation of injection drug use is part of the optimal holistic strategy for managing IE in IDU patients, it needs to be recognised that the expectation that this will be successful in all patients is unrealistic. Despite significant reforms in socioeconomic policies and increased funding to addiction medicine, substance use disorder is a complex medical condition and some patients will continue to inject drugs despite intervention. Our cohort shows that despite not requiring complete abstinence from drug use prior to IDU associated IE treatment, IDU patients still have better long-term outcomes compared to the non-IDU population, most likely due to this population being younger and relatively less comorbid at baseline. Therefore the mortality risk of injection drug use should not be overstated when significant protective factors are also prevalent in this population. We suggest guidelines should reflect these findings and that IDU patients should not have blanket restrictions on the management they are offered unless the individual has clear contraindications to a particular therapy that would also exclude a non-IDU patient.

## 5. Limitations

The findings of this study should be interpreted with regards to its single-centre observational cohort design. Although numerous risk factors were included in the analysis, the risk of confounding is present. Selection bias was minimised by including all patients admitted to our centre with the diagnosis of infective endocarditis, however as this is a single-centre study, the inner-city location of our tertiary centre may not be representative of the general population and represent a selection bias. Our study had a low lost-to-follow up rate, however some patients were still lost, therefore these patients may represent a population with a significant outcome or exposure that may affect our observed outcomes. Our database was cross-referenced with the ANZSCTS database to assess for reoperations at other centres, however early reoperations may have been missed if they occurred prior to the databases formation. Similarly, any reoperations that occurred in a different country would not have been included in our analysis.

## 6. Conclusion

Despite rising levels of IDU globally, there is a lack of clear guidelines on the management of IDU associated IE. After treatment, IDU patients are likely to continue to use drugs and are at a high risk of reinfection, which has led some authors to suggest restrictions on treatments offered to IDU patients. Our cohort shows that despite having statistically higher levels of reinfection and without mandatory abstinence from drugs, IDU patients still have better long-term outcomes compared to the non-IDU population, which is likely due to their younger age, lower level of comorbidity, and presentation at earlier stages in the disease course. Therefore we suggest that IDU patients should not have blanket restrictions on the management they are offered unless the individual has clear contraindications to a particular therapy that would also exclude a non-IDU patient.

## Supporting information

S1 Data (XLSX)
